# Genital Tuberculosis and Serous Cystadenoma in a 58-Year-Old Female With Rheumatoid Arthritis and Sjögren's Syndrome: A Case Report

**DOI:** 10.1155/crrh/9372058

**Published:** 2025-05-06

**Authors:** Sheila De la Cruz-Aragón, Itzel Guadalupe Castillo-Duarte, Abril Camacho-Cervantes, Alfredo Saad-Ganem, Francisco Mario García Rodríguez, Alan Antonio Leija-Torres

**Affiliations:** ^1^Department of Medicine, Universidad Nacional Autónoma de México, Ciudad de México, Mexico; ^2^Department of Obstetrics and Gynaecology, Hospital Español, Ciudad de México, Mexico; ^3^Department of Oncosurgery, Hospital Español, Ciudad de México, Mexico; ^4^Department of Medical Writing & Publications, BIPALT Consultoría Bioestadística, Ciudad de México, Mexico

**Keywords:** genital tuberculosis, ovarian neoplasms, rheumatoid arthritis, serous cystadenoma, Sjögren's syndrome, artritis reumatoide, cistadenoma seroso, neoplasias ováricas, síndrome de sjögren, tuberculosis genital

## Abstract

Genital tuberculosis (GT) is a rare but significant extrapulmonary tuberculosis form, often mimicking ovarian malignancy. We report a case of a 58-year-old woman with Sjögren's syndrome and rheumatoid arthritis, previously treated with infliximab, who presented with abdominal distension, weight loss, night sweats, and intermittent abdominal pain. Initial imaging and elevated CA-125 levels suggested ovarian cancer. However, intraoperative findings revealed a frozen pelvis with granulomatous inflammation, caseating granulomas, and Langhans' giant cells. Histopathological analysis and RT-PCR confirmed GT coexisting with a serous cystadenoma. GT should be considered in the differential diagnosis of pelvic masses, especially in immunocompromised patients. This case emphasizes the importance of thorough diagnostic evaluation using molecular, serological, and imaging techniques to avoid misdiagnosis and unnecessary surgical interventions. Prompt initiation of antituberculosis treatment led to significant clinical improvement. Early and accurate diagnosis of GT is crucial to prevent morbidity associated with misdiagnosis and to provide effective treatment. This case underscores the need for heightened clinical awareness and multidisciplinary approaches in managing complex cases where GT mimics malignancy, ensuring optimal patient outcomes.

## 1. Introduction

In 1744, an Italian anatomist Morgagni described the first case of genital tuberculosis (GT) in a 20-year-old female [1]. Tuberculosis (TB) is a disease prevalent worldwide. The actual incidence of pelvic/GT cannot be accurately assessed in any population since it is estimated that at least 11% of patients are asymptomatic, and the disease is discovered incidentally [1, 2]. The frequency varies considerably with geographic location. Female GT is a non-neoplastic disease that often resembles tumors affecting the female genital system in terms of clinical appearance and symptoms [3].

TB is currently a communicable disease and remains a major public health problem due to its global prevalence. Therefore, timely detection and treatment are crucial aspects to consider [2]. Misdiagnosis predisposes the patient to unnecessary surgery and medical treatment. Furthermore, there is an increase in economic burden and psychological stress for TB patients wrongly diagnosed as cancer patients [1, 3].

The diagnosis of female GT poses a diagnostic challenge. Hence, it is important to understand the natural history of the disease and the types of dissemination [4]. The different routes of dissemination are lymphatic, direct spread, and hematogenous, the latter being the most common type [5]. In some cases, primary female GT can rarely be transmitted to women whose male partners have active genitourinary TB, such as TB epididymitis, and transmits through infected semen [6–8]. In such cases, the vulva, vagina, or cervix are the first to get infected [9].

Some pharmacovigilance studies have shown evidence of an increased risk of extrapulmonary disease with monoclonal antibodies [10]. The frequency of extrapulmonary TB in patients treated with antitumor necrosis factor (TNF) therapy has ranged from 28% to 75%, with most reports indicating that over 50% of cases are extrapulmonary [10, 11]. In several clinical and experimental studies, when the containment of the bacteria in granulomas is obstructed by TNF-α neutralizing inhibitors, previously latent TB bacilli may cause disseminated TB disease [12]. GT demands immediate attention because of its low recovery rates and associated complications observed in recent years. Although this type of TB is a paucibacillary disease, the diagnosis is made through microbiological methods or a combination of tests [11].

Fortunately, the most effective treatment remains as anti-TB drugs for 4–6 months with high cure rates [5, 11, 13]. Several reviews have shown that treatment with these drugs can resolve symptoms within a week of starting the treatment [14].

We will discuss the case of a 58-year-old woman with a medical history of Sjögren's syndrome and rheumatoid arthritis. She was treated with infliximab (anti-TNF) for 6 months. The case involves the coexistence of female GT and an ovarian tumor that, in terms of clinical appearance and imaging, mimicked ovarian cancer.

## 2. Case Presentation

A 58-year-old female patient, G1P1 (Gravida 1, Para 1), presented to the gynecology outpatient clinic for an annual checkup. She reported a 2-month history of symmetrical abdominal distension, unintentional weight loss of 5 kg, night sweats, and episodes of constipation. She also experienced intermittent left lower quadrant abdominal pain.

She had a medical history of Sjögren's syndrome and rheumatoid arthritis, diagnosed 3 years prior, and treated with infliximab for 6 months. Her medication was switched to chloroquine and methotrexate 3 months before her visit. Surgical history included a myomectomy in 2003. Gynecological history revealed menarche at age 12, with her last menstrual period in 2019. Cervicovaginal cytology in 2023 was negative for malignancy as were for both breast ultrasound and mammography in 2022.

On physical examination, vital signs were normal. The abdomen displayed a suprapubic scar and was distended due to ascitic fluid. Decreased bowel sounds were noted. Tenderness was present in the left iliac fossa, but no palpable masses or signs of peritoneal irritation were found; percussion revealed dullness. Examination of external and internal genitalia was normal, and other systems were unremarkable.

Laboratory results indicated hypochromic microcytic anemia with a hemoglobin level of 11.5 g/dL (normal: 11.7–16.0 g/dL). Renal and liver function tests were normal, as was C-reactive protein. Serum tumor markers showed elevated CA-125 at 234 U/mL (normal: < 35 U/mL), while carcinoembryonic antigen and CA 19-9 levels were normal. Anti-HIV 1 and 2 antibody tests were nonreactive.

**Figure 1:**
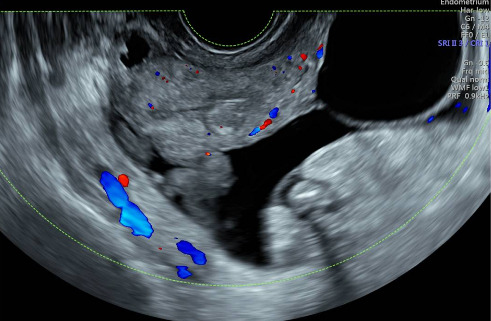
Transvaginal pelvic ultrasound showing a complex tumor dependent on the left ovary, with plenty of free peritoneal fluid in the pelvic cavity.

The chest X-ray was normal, but abdominal ultrasound revealed ascites and a suspicious left ovarian mass with multiloculated cysts and solid areas (Figure 1). The mass was categorized as O-RADS 5 (> 50% likelihood of malignancy, high risk), and the risk malignancy index was 2106 points, indicating a high risk of malignancy (71% sensitivity and 92% specificity for ovarian cancer) [15, 16].

**Figure 2:**
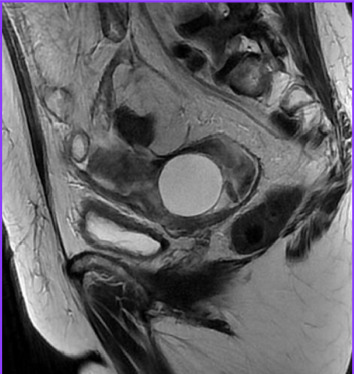
MRI T2 sequence, in a sagittal view, showing a complex tumor dependent on the left ovary.

**Figure 3:**
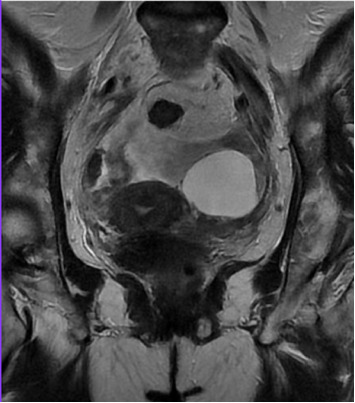
MRI T2 sequence, in a coronal view showing a complex tumor dependent on the left ovary.

MRI of the abdomen with intravenous contrast showed massive ascites and thickening of the parietal peritoneum, predominantly in the pelvis. Findings were inconclusive regarding malignancy or granulomatous entities. The left ovary exhibited a 4.1 cm simple cyst, and pelvic lymphadenopathy was noted (Figures 2 and 3).

**Figure 4:**
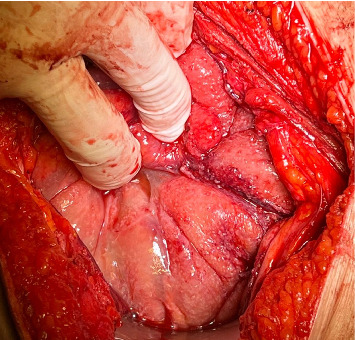
Laparotomy showing a frozen pelvis with granulomatous appearance of the pelvic peritoneum and bowel.

Based on the information collected, a laparotomy was proposed. Intraoperatively, a frozen pelvis was discovered, with the left ovary enlarged and surrounded by ascites. The pelvic peritoneum displayed thickening and a granulomatous appearance (Figure 4) with disseminated creamy brown patches. The liver, uterus, and right ovary were unremarkable. A biopsy of the pelvic parietal peritoneum was taken, and the left iliac nodes were removed. Approximately 450 mL of straw-colored ascitic fluid was collected. Intraoperative microscopic analysis by the hospital's pathology department revealed granulomatous inflammation with Langhans' giant cells. Consequently, a left salpingo-oophorectomy was performed.

This approach was chosen due to a strong clinical suspicion of ovarian carcinoma. According to the NCCN 2023 guidelines, laparotomy is the standard procedure for ovarian tumors, allowing for a comprehensive evaluation that includes peritoneal lavage, lymph node sampling, and tumor removal. Although minimally invasive surgery can minimize morbidity in diagnostic interventions, laparotomy was preferred in this case to ensure thorough assessment and management based on the intraoperative findings.

Lymph node sampling was performed as part of the standard ovarian cancer protocol. This procedure is crucial in assessing potential metastatic involvement and in providing additional diagnostic information when ovarian carcinoma is suspected.

The definitive histopathological analysis of the peritoneum biopsy revealed numerous caseating granulomas with Langhans' giant cells and abundant acid-fast bacilli (AFB), consistent with *Mycobacterium* TB. RT-PCR confirmed the diagnosis by detecting TB DNA. Similar findings were observed in the left ovarian cyst, where Ziehl–Neelsen staining confirmed the presence of AFB and the coexistence of a serous cystadenoma.

The patient started on anti-TB treatment with rifampicin and isoniazid, as well as pain medications. She had a good postsurgical recovery and was discharged on Day 3. Follow-up included monitoring CA-125 levels, which gradually decreased to normality.

## 3. Discussion

The patient started anti-TB treatment with rifampicin and isoniazid, as well as pain medications. She had a good postsurgical recovery and was discharged on Day 3. Follow-up included monitoring CA-125 levels, which gradually decreased to normal. GT is found in approximately 1% of all TB-positive patients [17, 18]. Its prevalence is highest in developing countries [19, 20], with up to a quarter of the world's population remaining dormant without clinical manifestations despite contact with the bacillus [20].

Although TB most frequently affects the lungs, it can also involve other organs, known as extrapulmonary TB. In women, the most common form is GT [11, 20]. Its presentation is atypical and clinically similar to other gynecological conditions, often misdiagnosed as ovarian malignancy [14]. Therefore, a palpable pelvic tumor mass should be considered in the differential diagnosis of GT due to its ambiguous and variable presentation, which can easily be confused with ovarian or endometrial cancer or peritoneal carcinomatosis [5, 8, 20]. Symptoms are related to the affected organs, with the most frequent being chronic pelvic pain, menstrual abnormalities, and infertility [4, 8]. Many cases may remain asymptomatic, with the diagnosis often being incidental through imaging studies [14].

GT typically occurs secondary to a primary lung infection [20] via hematogenous spread, which is the most common type of dissemination [20, 21]. Wagener et al. have also reported cases of sexual transmission and direct dissemination from surrounding organs [20] In rare cases, primary female GT can be transmitted to women whose male partners have active genitourinary TB, such as TB epididymitis, through infected semen [6–8]. In such cases, the vulva, vagina, or cervix is the first to get infected [9].

The most frequently affected organs are the fallopian tubes, up to 90% [4, 6, 21], usually presenting with hydrosalpinx, followed by endometrial and ovarian involvement [6, 21]. Our patient had ovarian and peritoneal involvement of the vulva and vagina is rare, occurring in 1%–2% of cases, presenting with hypertrophic lesions or recurrent genital ulcers [6, 8, 22]. GT should be suspected in cases of long-standing idiopathic infertility [4, 6] or chronic inflammatory pelvic disease unresponsive to conventional antibiotics [6].

Several factors promote infection, most being host-related and leading to deteriorated immunity [17]. Any acquired or innate immunodeficiency increases the risk of developing TB [18]. Other risk factors include low socioeconomic status, poor access to health services, malnutrition, diabetes, substance abuse, hemodialysis, smoking, and HIV infection, among others [6, 18]. Underlying immunosuppressive diseases favor the development of opportunistic infections such as TB [17]. The frequency of extrapulmonary TB in patients treated with anti-TNF therapy ranges from 28% to 75%, with most reports showing > 50% of cases as extrapulmonary [10, 11]. In addition, Dixon W. et al. have shown an increased risk of extrapulmonary disease with monoclonal antibodies [10, 11]. In our case, the patient had rheumatoid arthritis and anti-TNF therapy as risk factors, without contact with the bacilli and no associated respiratory symptoms.

TB physiopathology begins when bacilli infect a susceptible host. Upon entering the lungs, most bacilli are ingested by alveolar macrophages and dendritic cells and are isolated within granulomas. In granulomas, monocytes differentiate into epithelioid cells to form giant cells (Langhans cells). TNF-α is essential for forming and maintaining these cells. Clinical and experimental trials have shown that TNF-α neutralizing agents can disrupt bacterial containment in granulomas, leading to disseminated TB disease from previously latent bacilli [2, 12].

Rheumatic diseases are associated with a 2–4-fold increased risk. It has been reported that infliximab (a TNF-α neutralizer) treatment increases this risk by 8.9–30.1-fold [12]. Several inflammatory pathways have been identified as upregulated in endometrial biopsies of patients with GT and latent GT. These pathways include mitogen-activated protein kinase (MAPK), TNF, natural killer (NK) cells, toll-like receptors (TLRs) signaling, and nuclear factor kappa-B (NF-κB). Understanding these immune disturbances may help identify potential biomarkers [7, 11].

The diagnosis of GT is always histological, confirmed by a biopsy showing granulomatous infection, as in our case [20]. The diagnostic approach includes a complete blood count, renal and liver function tests, HIV 1 and 2 tests, Mantoux test, and interferon-gamma release assay (IGRA), along with CA-125 levels adjusted to the patient's menopausal status [8, 9, 20]. Specific diagnostic methods include imaging tests (chest X-ray, ultrasound, hysterosalpingography, CT, MRI, and PET), endometrial aspirate or biopsy (Ziehl–Neelsen stain, AFB culture, nucleic acid amplification test or GeneXpert, PCR, and epithelioid granuloma on histopathology) [8, 11, 13, 14], and endoscopic techniques (hysteroscopy and diagnostic laparoscopy) that allow direct visualization of TB lesions such as tubercles, caseous nodules, or adhesions/frozen pelvis, confirmed with biopsy samples [11, 22]. In our case, biopsies reported granulomatous inflammation with Langhans' giant cells and abundant AFB, consistent with *Mycobacterium* TB. The diagnosis was confirmed through RT-PCR for TB DNA detection.

In this case, imaging findings suggested an ovarian tumor with probable extension to the pelvic peritoneum of malignant vs. granulomatous origin. When TB involves the abdomen, it may present as tuberculous peritonitis with generalized or localized ascites and/or retroperitoneal lymphadenitis, as observed in our patient. Tuberculous peritonitis is observed in combination with GT in approximately 45% of cases [2]. It is important to consider Human Epididymis Protein 4 (HE4) in addition to CA-125 to differentiate between tuberculous infection and epithelial ovarian neoplasm. HE4 shows elevated levels in serous, endometrioid, and clear-cell ovarian neoplasms [14]. Joint evaluation of both biomarkers can help differentiate these pathologies preoperatively [14, 20]. Saini A. et al. have reported that TB can coexist with ovarian germ cell tumors, papillary serous carcinoma, and ovarian cystadenoma [14]. In our case, the patient had elevated CA-125 (234 U/mL) and coexisting serous cystadenoma.

Due to imaging findings and clinical suspicion, our patient was initially misdiagnosed with ovarian cancer. She underwent a staging laparotomy for histopathological diagnosis. Macroscopically, the peritoneal and pelvic cavities were atypical, showing caseous nodules, ascites, and a frozen pelvis with an enlarged left ovary. Intraoperative biopsies revealed granulomatous infection with Langhans' giant cells.

TB is a common cause of chronic pelvic inflammatory disease and infertility [23]. It should always be considered in the differential diagnosis of a pelvic mass, especially among immigrants from developing countries (Asia, the Middle East, and Latin America) and HIV-positive patients [18, 23, 24].

The treatment of GT follows a similar regimen to that of pulmonary TB, using four drugs: rifampicin, isoniazid, pyrazinamide, and ethambutol. These are administered orally daily for 2 months during the intensive phase, followed by three drugs taken orally daily for the subsequent 4 months during the continuation phase. Drug sensitivity testing is essential to identify cases of multidrug-resistant TB [11, 25].

In our case, the patient was treated with rifampicin. She completed treatment through the initial, intermediate, and final phases until November 2024, when she was discharged. During this period and to date, infliximab, chloroquine, and methotrexate were discontinued, and the patient remained on deflazacort 6 mg daily, initiated at diagnosis and continued thereafter.

It is important to note that rheumatological diseases inherently compromise a patient's immunity. When combined with monoclonal antibody treatment, this significantly increases the risk of opportunistic infections [26]. Thus, a comprehensive approach is necessary, and serial measurements of CA-125 can be valuable in monitoring the response to anti-TB medications [27].

## 4. Conclusion

This case highlights the need to consider a patient's rheumatological history and immunosuppressive treatment when evaluating adnexal masses with granulomatous features. Monoclonal antibody therapies, particularly TNF-α inhibitors, significantly increase the risk of TB reactivation, which can mimic ovarian malignancies and lead to misdiagnosis. Given the rising use of biologic therapies, clinicians should maintain a high index of suspicion for TB in patients presenting with pelvic masses, especially in endemic regions.

Women of reproductive age receiving monoclonal antibody therapy require careful assessment beyond routine imaging and tumor marker evaluation, as conventional diagnostic approaches may be insufficient in differentiating TB from malignancy. Enhanced awareness of these atypical presentations can facilitate earlier diagnosis, reducing unnecessary surgical interventions, emotional distress, and healthcare costs. By recognizing the association between monoclonal antibodies and TB risk, clinicians can improve patient outcomes through timely diagnosis and appropriate management.

## Data Availability

Data sharing is not applicable to this article as no datasets were generated or analyzed during the current study.
